# Total Hip Arthroplasty in Patients with BMI ≥ 30 kg/m^2^: BMI Evolution, Planning Accuracy, Restoration of Anthropometric Parameters and Clinical Outcomes

**DOI:** 10.3390/jpm16080395

**Published:** 2026-07-24

**Authors:** Costanza Martorelli, Alessandra Monzio Compagnoni, Matteo Massa, Gianluigi Pasta, Federico Alberto Grassi, Michela Saracco, Eugenio Jannelli

**Affiliations:** 1 Orthopedic and Traumatology Clinic, IRCCS Policlinico San Matteo Foundation, 27100 Pavia, Italy; costanzamartorelli01@universitadipavia.it (C.M.); a.monzio@smatteo.pv.it (A.M.C.); g.pasta@smatteo.pv.it (G.P.); f.grassi@smatteo.pv.it (F.A.G.); eugenio.jannelli@libero.it (E.J.); 2 Department of Clinical, Surgical, Diagnostic and Pediatric Sciences, University of Pavia, 27100 Pavia, Italy; 3 Department of Public Health, Orthopedic and Traumatology Clinic, University of Naples “Federico II”, 80131 Naples, Italy

**Keywords:** total hip arthroplasty, obesity, body mass index, anthropometric parameters, clinical outcomes

## Abstract

**Background:** Obesity is increasingly prevalent among patients undergoing total hip arthroplasty (THA). While outcomes are well reported, fewer studies assess postoperative BMI trends, digital templating accuracy, and biomechanical restoration. Purpose: To evaluate BMI evolution, templating accuracy, restoration of limb length and femoral offset, and clinical outcomes after primary THA in obese patients, compared with normal-weight controls. **Methods:** Retrospective comparative cohort including 75 obese patients (BMI ≥ 30 kg/m^2^) undergoing primary THA between July 2021 and December 2024. BMI was recorded preoperatively and at last follow-up. OrthoView™ 7.1 digital templating guided preoperative planning; accuracy was defined as implanted versus planned component sizes. Radiographic assessment of limb length and femoral offset was performed in 60 obese patients and 60 normal-weight controls. Clinical outcomes included Harris Hip Score (HHS), Oxford Hip Score (OHS), and EQ-5D-5L. Postoperative complications were recorded in the obese cohort. **Results:** BMI remained stable postoperatively (33.68 ± 2.57 vs. 34.06 ± 2.78 kg/m^2^; *p* = 0.138). Templating accuracy within ±1 size was high (femoral stem 80.2%; acetabular cup 94.7%). Limb-length discrepancy was low and comparable between obese and normal-weight patients (3.55 vs. 3.78 mm; *p* = 0.687). Femoral offset restoration was similarly comparable. Patient-reported outcomes improved significantly in both groups, with no significant differences in postoperative scores. **Conclusions:** In obese patients, THA provides reliable digital planning accuracy, satisfactory biomechanical reconstruction, and substantial functional improvement comparable to normal-weight patients. BMI remains essentially unchanged after surgery, indicating THA does not substantially influence postoperative weight trajectory in this population.

## 1. Introduction

Total hip arthroplasty (THA) is widely considered one of the most successful procedures in orthopaedical surgery, providing predictable pain relief, restoration of mobility, and improved quality of life for patients with advanced hip osteoarthritis [1]. Furthermore, other primary diagnoses, including avascular necrosis of the femoral head, congenital hip disorders, and inflammatory arthritis, frequently require surgical reconstruction. Continuous improvements in implant design, surgical techniques, and digital planning tools have contributed to increasingly reproducible outcomes.

In recent decades, the demographic profile of patients undergoing THA has progressively changed [2]. Obesity, defined by the World Health Organization as a body mass index (BMI) ≥ 30 kg/m^2^, is increasingly frequent among candidates for hip replacement [3]. Obese patients may present technical challenges related to soft-tissue envelope thickness and surgical exposure, raising concerns regarding implant positioning and biomechanical reconstruction and a potentially increased risk of perioperative and postoperative complications [4]. In these subjects, comorbidities, lifestyle habits, and altered anatomical landmarks significantly affect surgical approach, perioperative management, and functional recovery. Consequently, a meticulous preoperative evaluation is mandatory to optimize treatment customization and address the inherent difficulties of a more demanding surgical exposure and prolonged operative times.

Accurate restoration of limb length and femoral offset is essential to optimize hip biomechanics, abductor function, and postoperative stability [5]. While numerous studies have analyzed complication rates and implant survivorship in obese patients, fewer investigations have specifically evaluated planning accuracy and restoration of anthropometric parameters in this population [6,7].

The purpose of the present study was therefore to analyze BMI evolution, digital templating accuracy in identifying the correct implant sizes, and restoration of limb length and femoral offset. Furthermore, we aimed to evaluate the postoperative clinical outcome using specific validated questionnaires, namely the Harris Hip Score (HHS), Oxford Hip Score (OHS), and EQ-5D-5L, comparing these findings to a normal-weight control group. We hypothesized that accurate biomechanical reconstruction and comparable clinical improvement could be achieved despite increased BMI.

## 2. Materials and Methods

### 2.1. Study Design and Patient Selection

This retrospective comparative cohort study was conducted at the Orthopaedics and Traumatology Department of IRCCS Policlinico San Matteo Foundation (Pavia, Italy). All obese patients (BMI ≥ 30 kg/m^2^) undergoing primary THA with a posterolateral approach between July 2021 and December 2024 were considered eligible following a diagnosis of hip osteoarthritis or aseptic necrosis of the femoral head. The minimum follow-up was 12 months, the maximum 54 months with a mean follow-up of 31.7 months.

Inclusion criteria were age ≤ 86 years, diagnosis of hip osteoarthritis or femoral head osteonecrosis and availability of preoperative digital templating and follow-up radiographs. Among 93 eligible patients, 17 were lost to follow-up and one died before study completion, resulting in a final cohort of 75 obese patients. The final study population comprised 28 female patients (37.3%) and 47 male patients (62.7%), with a mean age of 65.11 ± 9.97 years (range: 38–86 years). Throughout the study period, a progressively increasing number of surgical procedures was recorded: 7 interventions were performed in 2021, 19 in 2022, 31 in 2023, and 18 in 2024. 

In addition to baseline characteristics, specific risk factors and comorbidities were recorded and analyzed, including smoking status, diabetes, and advanced age (defined as age > 70 years). Smoking status, diagnosis of diabetes and the use of antihyperglycemic oral medications or insulin administration were collected from the past medical history of the patient. The inclusion of these variables was motivated by their potential influence on the postoperative outcomes of patients undergoing total hip arthroplasty. The objective of this analysis was twofold: first, to evaluate the prevalence of these comorbidities within the studied sample; and second, to investigate any potential statistical association between their presence and the occurrence of early or late postoperative complications.

A control group of 75 normal-weight patients treated during the same period, and then with homogeneous follow-up, using the same surgical approach, was selected for comparison of clinical and radiographic outcomes. The control group was also similar in terms of sex distribution, age and smoke habits.

To assess postoperative restoration of anthropometric and biomechanical parameters, from July 2022 a subgroup of obese patients (*n* = 60) and an equal number of normal-weight controls (*n* = 60) underwent standardized postoperative weight-bearing AP pelvis radiographs with calibration marker. A postoperative “re-templating” was then performed using OrthoView™ 7.1 (Meridian Technique Ltd., Southampton, UK), adopting the same anatomical landmarks used during preoperative planning to evaluate limb length and femoral offset.

All preoperative planning procedures and radiographic evaluations were performed by a single experienced orthopedic surgeon specialized in hip arthroplasty. It should be noted that the evaluator was not necessarily the surgeon who performed the surgical procedure. This approach was adopted to ensure methodological uniformity and to minimize potential bias related to preoperative planning and radiographic assessment between the two study groups.

### 2.2. Surgical Technique

**Figure 1:**
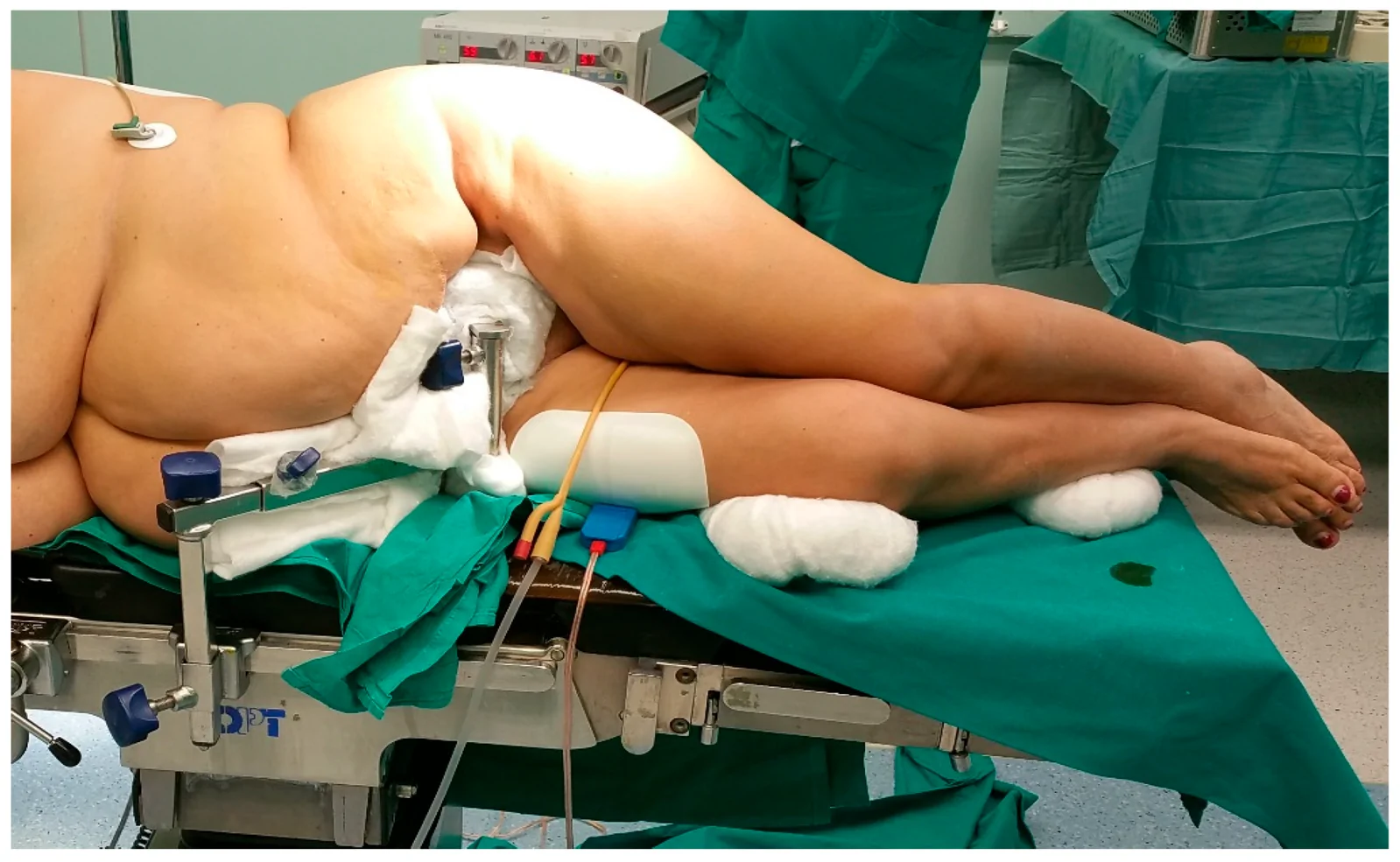
Patient surgical positioning.

All procedures were performed under spinal anesthesia through a standardized posterolateral approach with the patient in lateral decubitus (Figure 1). Cementless acetabular and femoral components were implanted in all cases.

After trial reduction, hip stability, limb length and offset were assessed intraoperatively. Attention was given to verifying the biomechanical reference parameters identified during the preoperative planning. Final components were implanted only after confirming satisfactory stability and restoration of the planned measurements.

### 2.3. Radiographic Evaluation

Standardized anteroposterior pelvis radiographs were obtained preoperatively and postoperatively with the beam centered on the pubic symphysis and both femurs positioned in 15–20° of internal rotation to parallel the natural femoral anteversion. To ensure an accurate frontal and sagittal pelvic alignment, radiographs were evaluated for symmetry, ensuring the pubic symphysis projected directly over the center of the sacrum. Anteroposterior pelvis radiographs were repeated 3 months after surgery using a calibration marker to allow accurate digital measurements. Radiographic analysis was performed using OrthoView™ 7.1—2017 (Meridian Technique Ltd., Southampton, UK). Magnification remains a critical variable in digital templating, typically averaging 20% assuming a standard tube-to-table distance of 1 m and film placement 5 cm beneath the table. Since radiographic magnification is directly proportional to the distance between the pelvis and the film, a significantly higher magnification factor is expected in obese patients compared to lean individuals. Therefore, a high-precision calibration marker of known diameter was positioned at the level of the joint to standardize and correct all digital measurements.

Limb-length discrepancy (LLD) was assessed on standardized anteroposterior pelvic radiographs using a horizontal reference line tangent to the inferior margins of the ischial tuberosities (bi-ischial line). A horizontal line was drawn connecting the most inferior points of the ischial tuberosities. The vertical distance between this line and the most prominent point of the lesser trochanter was measured for each femur. The difference between the two sides was recorded as limb-length discrepancy. Femoral offset was defined as the perpendicular distance between the femoral head center and the femoral shaft axis. Offset variation was calculated as the difference between preoperative and postoperative measurements [8,9].

### 2.4. Templating Accuracy

**Figure 2:**
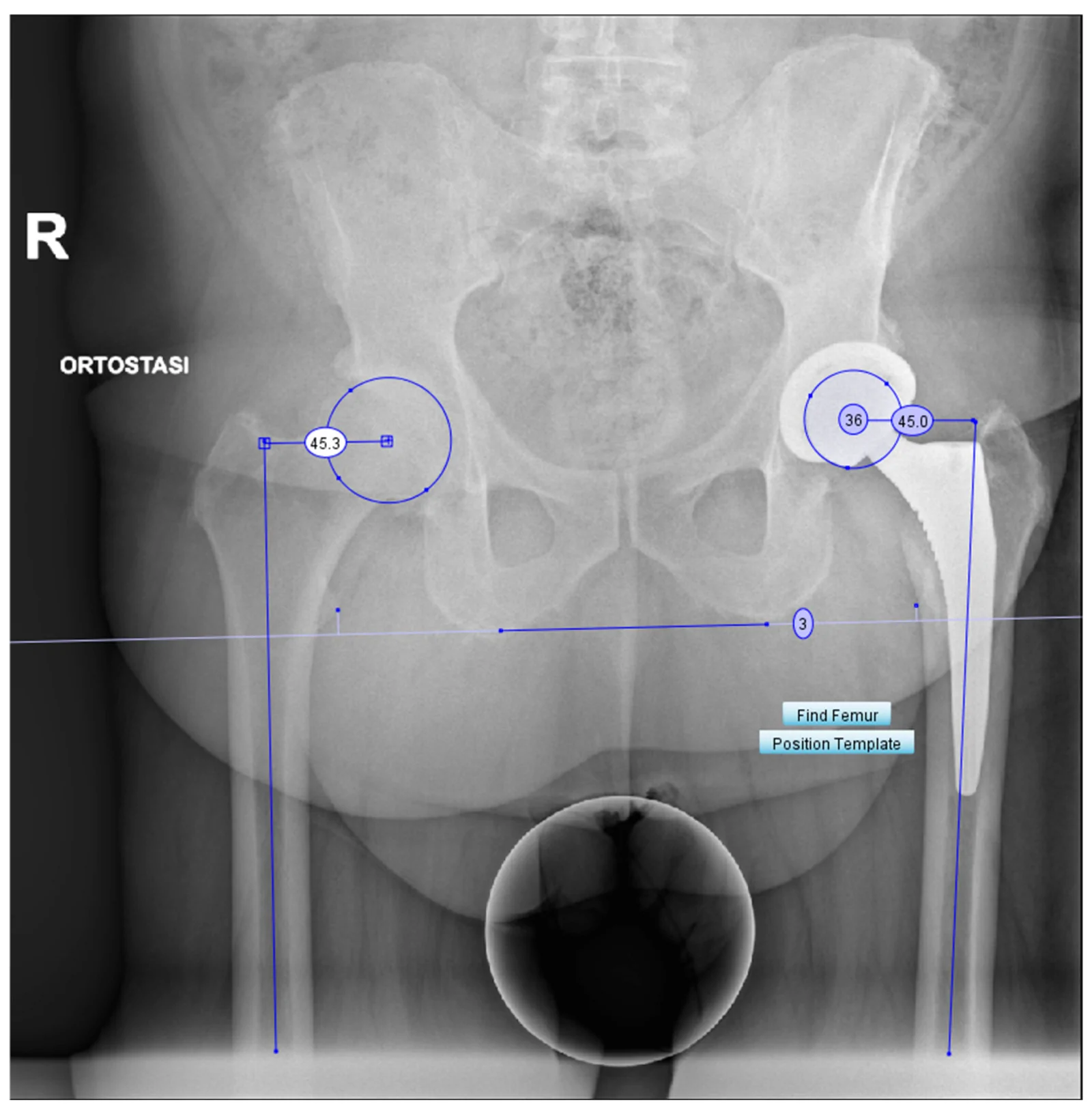
Pre-operative planning on an AP pelvis X-Ray performed in standing position: a horizontal line was drawn connecting the most inferior points of the ischial tuberosities. The vertical distance between this line and the most prominent point of the lesser tro-chanter was measured for each femur to assess the LLD; Femoral offset was defined as the perpendicular distance between the femoral head center and the femoral shaft axis.

Preoperative digital templating was performed using OrthoView™ 7.1—2017 (Meridian Technique Ltd., Southampton, UK) (Figure 2).

Planned component sizes were compared with the components implanted during surgery. Planning accuracy was evaluated both as absolute concordance and concordance within ±1 size. To evaluate the accuracy of biomechanical reconstruction, the subgroup of 60 obese patients and 60 normal-weight controls underwent standardized weight-bearing AP pelvis radiographs postoperatively. A rigorous ‘re-templating’ procedure was then performed using OrthoView™ 7.1 software, utilizing the exact same anatomical landmarks mapped during the preoperative phase. This allowed a precise comparative analysis of the actual restoration of limb length, femoral offset, and neck-shaft angle (ranging between 127° and 132° to optimize gluteal muscle tension) between the two cohorts.

### 2.5. Clinical Evaluation

Clinical outcomes were systematically assessed preoperatively and at the final follow-up using three validated patient-reported outcome measures (PROMs).

The Harris Hip Score (HHS) was utilized as a multidimensional instrument to evaluate the patient’s symptoms, health status, and functional capacity. The score comprises specific sections evaluating pain severity, gait capacity, activities of daily living (ADLs), deformity, and hip joint range of motion (ROM). The final cumulative score ranges from 0 (maximum disability) to 100 (absence of disability). Postoperative clinical results were categorized as excellent (90–100), good (80–89), fair (70–79), or poor (less than 70).

The Oxford Hip Score (OHS), a patient-centered questionnaire specifically designed to assess disability in patients undergoing total hip arthroplasty, was also administered. It consists of 12 items focusing on joint pain and physical capabilities experienced by the patient over the preceding 4 weeks. The total score ranges from 0 to 48, where 48 represents optimal joint function. Clinical outcomes based on the OHS were classified as excellent (greater than 41), good (34–40), fair (27–33), or poor (less than 27).

Health-related quality of life (HRQoL) and cost-effectiveness profiles were evaluated using the EQ-5D-5L questionnaire. This tool includes a five-dimension descriptive system (mobility, self-care, usual activities, pain/discomfort, and anxiety/depression), with each dimension scored across 5 levels of severity. The resulting health state is expressed as a 5-digit code ranging from 11,111 (indicating no problems in any domain) to 55,555 (indicating extreme problems across all dimensions). A country-specific value set algorithm was subsequently applied to convert these 5-digit health states into a single utility index score. Additionally, the EQ Visual Analogue Scale (EQ VAS) was used to record the patients’ self-rated health on a vertical, visual scale ranging from 0 (worst imaginable health state) to 100 (best imaginable health state).

Clinical outcomes were assessed using the Harris Hip Score (HHS), Oxford Hip Score (OHS) and EQ-5D-5L.

Scores were collected for both preoperative condition and final follow-up.

### 2.6. Statistical Analysis

Normality of continuous variables was assessed using the Shapiro–Wilk test. Given the sample size (greater than or equal to 30 subjects per group) and the robust nature of parametric tests against moderate violations of normality, between-group comparisons were performed using independent-samples *t*-tests. In the presence of non-homogeneous variances, determined by Levene’s test value of *p* < 0.05, Welch’s corrected version of the *t*-test was applied.

Within-group comparisons were performed using paired *t*-tests and between-group comparisons using independent-samples *t*-tests. Statistical significance was set at *p* ≤ 0.05. Analyses were conducted using STATA (release 10.0, StataCorp, College Station, TX, USA). Quantitative continuous variables are presented as mean ± standard deviation (SD), while qualitative categorical variables are expressed as absolute frequencies and percentages.

## 3. Results

**Figure 3:**
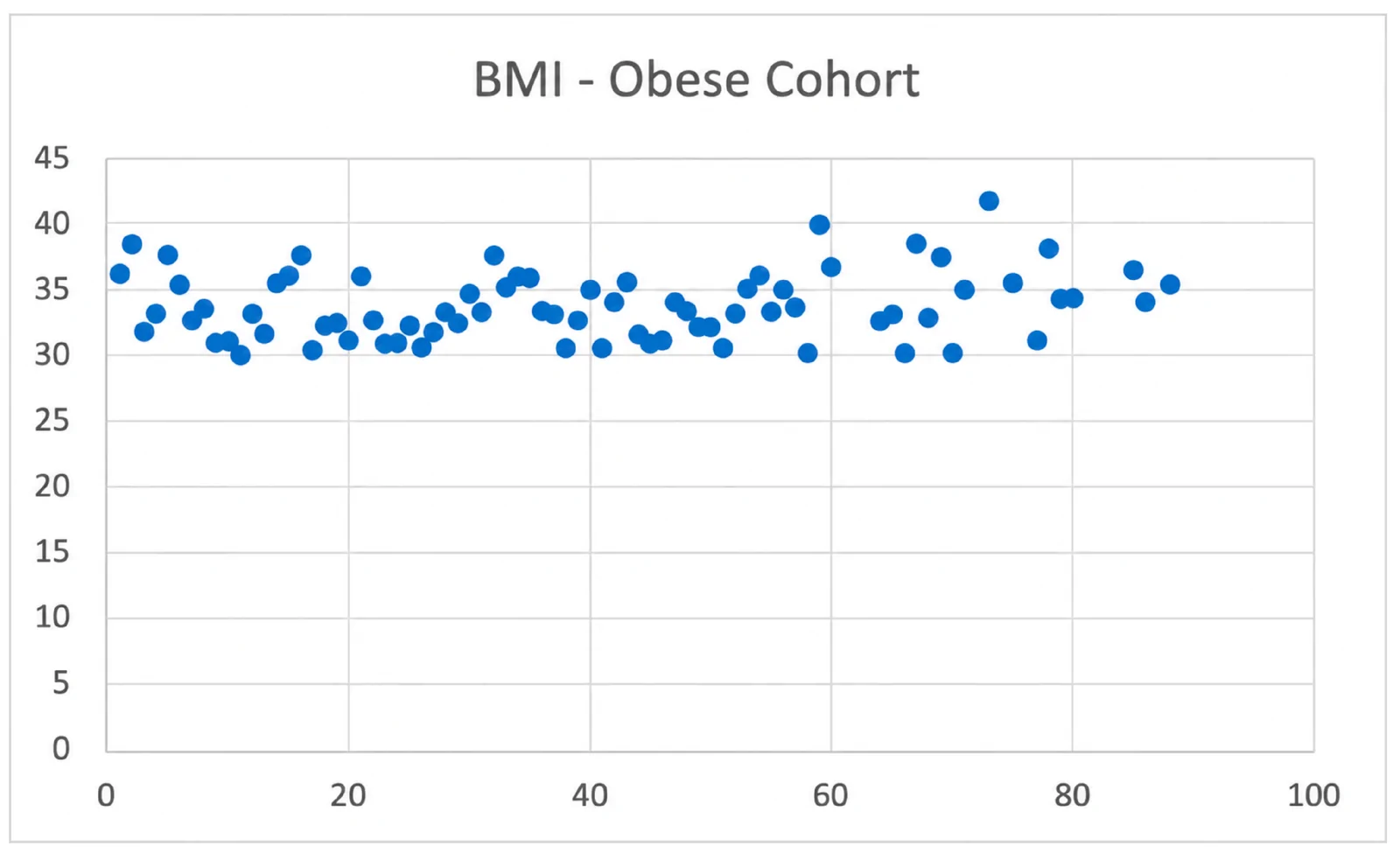
BMI distribution among the obese group.

A total of 75 obese patients undergoing primary THA were included in the study. All patients completed the preoperative assessment, and all were available at final follow-up, although 6 patients experienced postoperative complications. Three clinically relevant comorbidities were specifically analyzed within the obese cohort: 27 patients (36%) were older than 70 years, 28 patients (37.3%) had diabetes mellitus, and 14 patients (18.7%) were active smokers. The mean BMI in the obese cohort was 33.68 ± 2.57 kg/m^2^ preoperatively and 34.06 ± 2.78 kg/m^2^ at final follow-up, with no statistically significant difference between the two measurements (*p* = 0.138). Figure 3 reports the BMI distribution among the obese cohort of patients.

Digital preoperative templating demonstrated good agreement with the components implanted during surgery. Exact concordance between planned and implanted size was observed in 40.8% of femoral stems and 54.7% of acetabular cups. When considering concordance within ±1 size, templating accuracy increased to 80.2% for femoral stems and 94.7% for acetabular cups. Femoral head size prediction showed similar accuracy, with concordance within ±1 size in 80.4% of cases. For femoral stems, implanted size was smaller than planned in 33.8% of patients and larger in 25.3%, although most discrepancies involved only one size difference. Regarding acetabular components, implanted cup size was smaller than planned in 30.6% of cases and larger in 14.7%, while discrepancies greater than one size occurred only rarely. Similar findings were observed for femoral head components. These results confirm good overall reliability of digital templating despite the intrinsic limitations of two-dimensional planning (Table 1).

Radiographic evaluation showed satisfactory restoration of anthropometric parameters. Mean postoperative limb-length discrepancy was 3.55 mm in obese patients and 3.78 mm in normal-weight patients.

**Figure 4:**
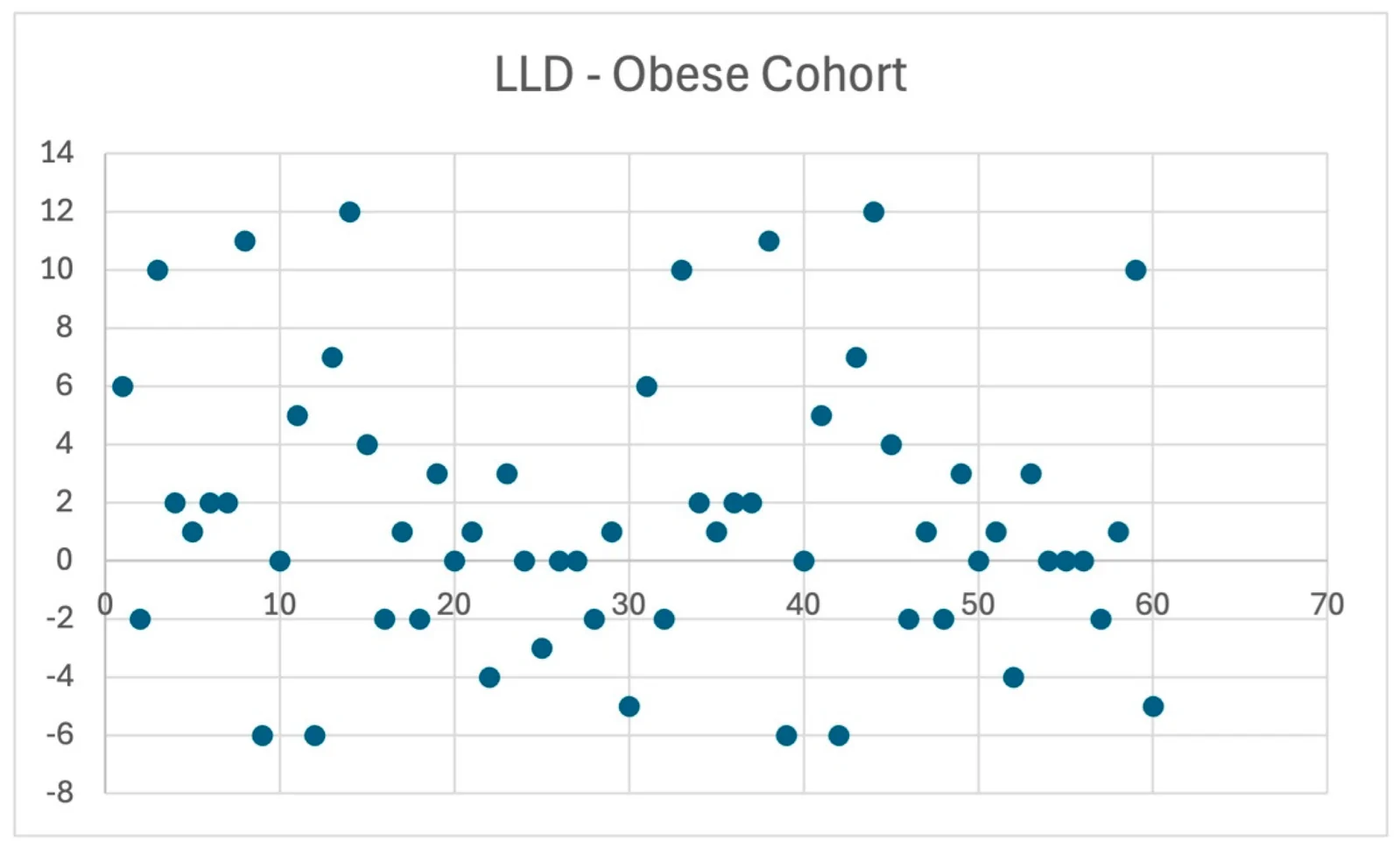
LLD distribution among the obese group of patients.

**Figure 5:**
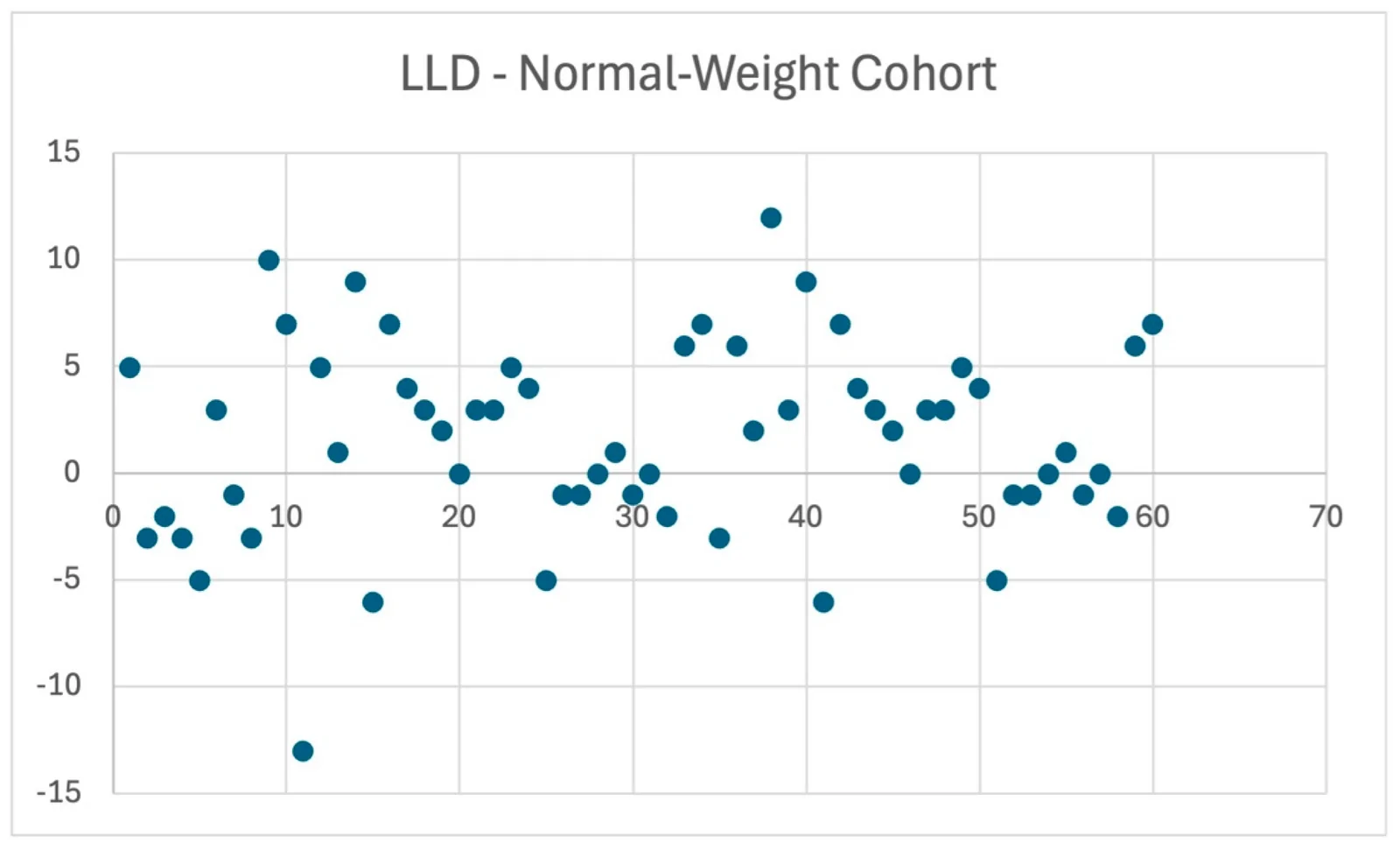
LLD distribution among the control normal-weight group.

Figure 4 and Figure 5 report the LLD values among the obese group and the control group respectively. Femoral offset variation was similarly small in both groups. The average postoperative change in obese patients is 2.70 mm and is even lower than that of normal-weight patients (4.06 mm). Although the difference reached statistical significance (*p* = 0.020), offset restoration remained within clinically acceptable limits in both cohorts. (Table 1) Femoral offset restoration was similarly satisfactory in both cohorts. The average postoperative offset variation in obese patients was 2.70 mm, which was significantly smaller than that observed in normal-weight patients (4.06 mm; *p* = 0.020). Obese patients more frequently demonstrated offset restoration within the 0–5 mm category and showed fewer marked deviations (>10 mm), suggesting even more accurate offset reconstruction compared with the control group (Table 2). 

Clinical outcomes improved significantly after surgery in both groups (Table 3).

The Harris Hip Score (HHS) increased from a preoperative mean value of 32.01 to 79.46 at final follow-up in the obese cohort (*p* < 0.001), corresponding to a mean increase of 47.45 points, indicating a substantial improvement in pain relief and hip function. Preoperatively, all obese patients presented poor HHS values (<70 points). Approximately 40% of patients achieved an HHS improvement greater than 50% following surgery, indicating substantial perceived clinical benefit.

Smoking status significantly influenced HHS improvement. Non-smokers demonstrated greater postoperative HHS gains compared with smokers (48.94 vs. 40.97 points; *p* = 0.027). No statistically significant differences in HHS improvement were observed according to diabetes status or age > 70 years.

The Oxford Hip Score (OHS) improved from a mean preoperative value of 21.16 to 38.29 postoperatively (*p* < 0.001), reflecting a marked improvement in patient-perceived hip-related quality of life. Approximately 20% of patients showed an improvement greater than 50% in OHS. No statistically significant associations emerged between OHS improvement and smoking status, diabetes, or age.

Similarly, the EQ-5D-5L index score increased from 0.47 preoperatively to 0.86 at final follow-up (*p* < 0.001), indicating a significant improvement in overall health-related quality of life. Mean EQ-5D-5L improvement was 0.39 points. At final follow-up, 20% of patients reported a state of complete health (EQ-5D-5L = 1), while all patients reported postoperative values above 0.5. Additionally, 29.3% of patients demonstrated an increase greater than 50% in EQ-5D-5L score. No statistically significant correlations were found between EQ-5D-5L improvement and smoking, diabetes, or age.

When compared with the normal-weight control group, postoperative PROMs did not show statistically significant differences between groups, suggesting comparable functional recovery and patient satisfaction following THA regardless of BMI category.

Postoperative complications in obese patients were recorded in 6 patients (8%): 2 patients (2.67%) required blood transfusions during hospitalization, 2 patients (2.67%) developed an infection at 35 and 77 days after surgery and 2 patients (2.67%) had a posterior implant dislocation occurring 69 and 85 days after surgery. No post-operative complications were recorded in the normal-weight cohort of patients. No further adverse events were observed during follow-up.

## 4. Discussion

Total hip arthroplasty (THA) is widely recognized as one of the most effective surgical procedures for relieving pain and restoring function in patients with advanced hip disease. However, despite its excellent clinical efficacy, THA is also associated with substantial economic burden for healthcare systems and an increasing number of revision procedures worldwide. The demand for THA is expected to further increase in the aging population due to the growing prevalence of major risk factors such as obesity and osteoarthritis. Moreover, both hip osteoarthritis and femoral head osteonecrosis are more frequently observed in male patients, with osteonecrosis showing a particularly marked male predominance.

The present study evaluated BMI evolution, accuracy of digital preoperative templating, restoration of anthropometric parameters, and clinical outcomes after primary total hip arthroplasty (THA) in obese patients, with comparison to a normal-weight control group. The main findings were that BMI remained stable after surgery, digital templating demonstrated good accuracy in obese patients, restoration of limb length and femoral offset was satisfactory and comparable to normal-weight individuals, and obese patients achieved substantial functional improvement with postoperative PROMs like those observed in non-obese patients.

Body weight remained essentially unchanged after surgery. This finding is consistent with previous studies reporting that spontaneous weight reduction after THA is uncommon despite improved mobility and pain relief [10]. Wu et al. [11] showed that most obese patients maintain stable BMI following THA, while Tidd et al. [10] reported minimal influence of perioperative weight change on postoperative outcomes. These findings suggest that THA should be considered a procedure aimed at restoring joint function rather than a strategy for weight reduction. Although slight postoperative BMI reduction has occasionally been described in obese patients, clinically significant weight loss is uncommon within the first postoperative year and may become evident only after longer follow-up periods. In severely obese patients, longer follow-up may be required to observe clinically relevant changes in body weight [11].

Digital templating demonstrated good reliability in this cohort. Accuracy within ±1 component size was comparable to values reported in large contemporary series evaluating digital planning systems. Zampogna et al. [12] reported similar templating accuracy in uncemented THA, confirming the reliability of modern digital planning tools. Importantly, our results suggest that elevated BMI does not substantially compromise templating accuracy when standardized radiographic techniques and calibration markers are used. These findings support the role of standardized digital templating as a reliable tool even in patients with elevated BMI, while also reflecting the intrinsic limitations of 2D planning and radiographic variability [12,13,14].

Restoration of limb length and femoral offset was satisfactory and comparable between obese and normal-weight patients. Thresholds commonly considered acceptable are <10 mm, with ≤5 mm often reported as an optimal target [15]. In the present study, mean postoperative LLD was low (approximately 3.5–3.8 mm) and did not differ significantly between obese patients and the normal-weight control group, suggesting that obesity did not compromise limb-length restoration in this setting. Conflicting results have been reported regarding the effect of BMI on limb-length control; some studies observed an association between higher BMI and less accurate restoration, whereas others, particularly when standardized techniques are applied, found limited or no impact [16,17].

Restoration of femoral offset is biomechanically important to re-establish abductor lever arm, optimize soft-tissue tension, enhance stability, and support physiological load distribution after THA [16,17]. In this study, the average offset variation was smaller in obese patients than in normal-weight controls (2.70 mm vs. 4.06 mm; *p* = 0.02) which may reflect increased attention to stability and abductor leverage during reconstruction in obese patients.

According to the current literature, the reported incidence of postoperative dislocation following primary total hip arthroplasty ranges from approximately 1% to 4%, and the incidence observed in our study is therefore consistent with previously published data. Specifically, the dislocation cases reported in our study exhibited leg length discrepancies of +10 mm and +5 mm, respectively, and femoral offset differences of −3.0 mm and −3.8 mm, respectively. These findings suggest a possible association; however, given the small sample size, it is difficult to establish a clear correlation.

Although counterintuitive given the technical challenges potentially associated with elevated BMI, previous prospective work suggests that BMI may affect some reconstruction parameters (particularly LLD) while having a more limited effect on offset restoration and cup positioning when surgical technique and planning are standardized [16,17]. A cautious interpretation of this finding is warranted: the magnitude of difference was moderate, and the radiographic subgroup size (60 patients per group) may limit the robustness of between-group inferences.

PROMs improved substantially after THA in obese patients. Harris Hip Score (HHS) increased from 32.01 to 79.45, Oxford Hip Score (OHS) from 21.16 to 38.29, and EQ-5D-5L index from 0.47 to 0.86. The observed postoperative HHS values are in line with literature showing marked improvements after THA, although many series report higher absolute postoperative values at 1–2 years, often in the 80–90+ range [18,19,20]. The relatively lower absolute postoperative HHS in this cohort may plausibly reflect the follow-up window (minimum 1 year), as functional recovery may continue beyond the first postoperative year [18,19,20].

For OHS, improvements of a clinically meaningful magnitude are expected after THA, with postoperative values frequently approaching 40–41 within the first year [21,22,23]. The present findings are consistent with these sources. Prior evidence suggests that obese patients may experience slightly smaller gains or lower absolute PROM values than normal-weight patients, while still achieving clinically significant improvement [24,25,26]. In this series, the normal-weight control group showed a slightly greater OHS improvement, statistically significant but of moderate magnitude, consistent with registry-based observations [25,26].

EQ-5D-5L typically increases after THA with a clinically relevant change, and large registries show meaningful improvements across BMI categories [26,27,28]. In the present study, obese patients showed high pre and postoperative EQ-5D-5L values and a comparable ΔEQ-5D-5L between obese and normal-weight patients, suggesting a similar relative benefit from surgery. Differences in absolute EQ-5D-5L levels can be influenced by extra-articular factors (e.g., psychosocial context, psychological condition, expectations, other musculoskeletal pain), which may modulate generic health measures independently of hip-specific improvement [28,29].

In this series, the overall complication rate in the obese cohort was 8%, with surgical site infection and posterior dislocation each occurring in 2.7% of patients. These events occurred within the early postoperative period (5–12 weeks for dislocation and 5–11 weeks for infection), which is consistent with the typical temporal pattern reported for early wound-related complications and instability after THA.

In the literature, obesity is commonly associated with an increased risk of postoperative complications compared with normal-weight patients, particularly for wound complications/infection and, in some series, instability and revision. Large observational studies and meta-analyses evaluating outcomes stratified by BMI generally report higher complication rates in obese and especially morbidly obese patients, while still confirming clinically meaningful functional improvement after THA. Accordingly, the complication profile observed in the present obese cohort appears broadly consistent with published evidence describing obesity as a risk modifier rather than an absolute contraindication to expected benefit [24,25]. These findings reinforce the concept that obesity should be regarded as a risk modifier rather than a contraindication to primary THA. Although obese patients may present increased technical complexity and potentially higher complication rates, the functional benefits achieved after surgery appear comparable to those observed in normal-weight individuals.

This study has several limitations. First, its retrospective design introduces potential selection bias. Second, the sample size was relatively limited, and BMI was not stratified into obesity classes, without distinction between lean mass and fat mass, making it impossible to determine whether postoperative weight variations reflected metabolic worsening or muscle recovery. Another important limitation was the absence of data regarding postoperative diet and physical activity, which may have influenced BMI evolution and quality-of-life outcomes. Third, anthropometric measurements were based on plain radiographs, which may be influenced by patient positioning and radiographic variability.

The statistical analysis did not reveal any significant differences between the two groups, although it is reasonable to hypothesize that patients with obesity would have poorer outcomes. This finding should be interpreted in light of several study-related factors. Although participants in the obesity group met the diagnostic criteria for obesity (BMI ≥ 30 kg/m^2^), their mean BMI was 33.7 kg/m^2^, reflecting a relatively moderate degree of obesity. Consequently, the difference in BMI between the two groups may not have been sufficiently large to produce measurable differences in the outcomes assessed. Furthermore, the relatively small sample size may have limited the statistical power of the study, increasing the risk of a type II error and reducing the ability to detect true between-group differences. Therefore, the combination of a relatively narrow BMI range and the limited sample size may have contributed to the absence of statistically significant findings. Future studies including larger cohorts and participants with a broader BMI spectrum, particularly those with more severe obesity, are warranted to better elucidate the impact of BMI on the variables investigated.

Overall, the present findings contribute to the growing body of evidence supporting equitable access to THA regardless of BMI. Obesity alone should not be considered an exclusion criterion for primary THA, but rather a perioperative risk factor requiring careful optimization and complication management. Furthermore, these results may help refine future patient selection criteria and perioperative management strategies for obese individuals undergoing THA.

## 5. Conclusions

In obese patients undergoing primary THA, digital templating demonstrated good accuracy and restoration of limb length and femoral offset was satisfactory. Functional outcomes improved substantially after surgery and were comparable to those observed in normal-weight patients. These findings support the role of standardized planning and careful intraoperative verification of biomechanical parameters when performing THA in patients with elevated BMI. Therefore, obesity does not impair the effectiveness of total hip arthroplasty in terms of functional recovery or improvement in quality of life. Normal-weight patients achieve slightly greater OHS improvement, although final outcomes are comparable to those of obese patients. Obese patients show higher absolute EQ-5D scores, with similar relative benefits. Leg length discrepancy and offset are adequately restored in both groups, with offset restoration being even more accurate in obese patients. These findings support existing evidence that THA provides substantial clinical benefit also in obese patients, and that BMI alone should not be considered an exclusion criterion, but rather a risk factor to account for in complication management rather than in the prediction of functional outcomes. Furthermore, these results contribute to the growing body of evidence supporting equitable access to THA regardless of BMI and may help refine patient selection criteria and perioperative management strategies in future literature.

## Figures and Tables

**Table 1:** Components of implants.

Component	Exact Match (%)	±Size (%)
Femoral stem	40.8	80.2
Acetabular cup	54.7	94.7
Femoral head	40.9	80.3

**Table 2:** Length and Offset of implants.

	Category.	Obese Patients (*n* = 60)	Normal Weight (*n* = 60)	Average Obese	Average Normal-Weight	*p*-Value (Welch *t*-Test)
Δ Lenght	0–5 mm	45	45	3.55	3.78	0.687
	5–10 mm	11	13			
	>10 mm	4	2			
Δ Offset	0–5 mm	48	37	2.70	4.06	0.020
	5–10 mm	10	18			
	>10 mm	2	5			

**Table 3:** Proms in the obese patients and in the normal weight patients.

	Average Obese	Average Normal Weight	SD Obese	SD Normal Weight	*p*-Value
HHS pre	32.01	29.61	12.48	11.45	0.222
HHS post	79.46	80.95	14.72	13.45	0.519
HHS diff	47.45	51.34	12.3	14.62	0.08
OHS pre	21.16	20.84	6.58	6.29	0.761
OHS post	38.29	40.57	9.53	5.75	0.079
OHS diff	17.13	19.73	7.26	6.75	0.02
EQ-5D-SL pre	0.47	0.33	0.22	0.2	0.00005
EQ-5D-SL post	0.86	0.77	0.1	0.13	0.00002
EQ-5D-SL diff	0.39	0.44	0.19	0.21	0.157

## Data Availability

The original contributions presented in this study are included in the article. Further inquiries can be directed to the corresponding author.
